# Moving beyond the desktop: prospects for practical bioimage analysis via the web

**DOI:** 10.3389/fbinf.2023.1233748

**Published:** 2023-07-25

**Authors:** Wei Ouyang, Kevin W. Eliceiri, Beth A. Cimini

**Affiliations:** ^1^ Science for Life Laboratory, Department of Applied Physics, KTH Royal Institute of Technology, Stockholm, Sweden; ^2^ Morgridge Institute for Research and Center for Quantitative Cell Imaging, University of Wisconsin-Madison, Madison, WI, United States; ^3^ Imaging Platform, Broad Institute of MIT and Harvard, Cambridge, MA, United States

**Keywords:** bioimage analysis, browser computing, cloud computing, web-based tools, data management, collaboration in science, data accessibility and reusability

## Abstract

As biological imaging continues to rapidly advance, it results in increasingly complex image data, necessitating a reevaluation of conventional bioimage analysis methods and their accessibility. This perspective underscores our belief that a transition from desktop-based tools to web-based bioimage analysis could unlock immense opportunities for improved accessibility, enhanced collaboration, and streamlined workflows. We outline the potential benefits, such as reduced local computational demands and solutions to common challenges, including software installation issues and limited reproducibility. Furthermore, we explore the present state of web-based tools, hurdles in implementation, and the significance of collective involvement from the scientific community in driving this transition. In acknowledging the potential roadblocks and complexity of data management, we suggest a combined approach of selective prototyping and large-scale workflow application for optimal usage. Embracing web-based bioimage analysis could pave the way for the life sciences community to accelerate biological research, offering a robust platform for a more collaborative, efficient, and democratized science.

## 1 Introduction

Bioimage analysis has become an indispensable tool in modern biological research, playing a crucial role in advancing our understanding of complex biological systems. The rapid development of imaging instrumentation has resulted in the generation of massive image data sets, capturing information from the molecular to the organismal level (Hallou et al., 2021; Chandrasekaran et al., 2023). Concurrently, advances in artificial intelligence (AI) and computer vision have revolutionized bioimage analysis by enabling the automated extraction of quantitative data from these large-scale image collections (Ravindran, 2022). Furthermore, bioimage analysis has become integral to spatial multi-omics research, where the integration of imaging data with transcriptomics, proteomics, and metabolomics data allows for a more comprehensive understanding of biological systems (Lundberg and Borner, 2019).

Despite the significant advancements in bioimage analysis, researchers still face challenges when it comes to collaboration, data sharing, and the implementation of diverse analysis approaches (Soltwedel and Haase, 2023). The ever-increasing size and complexity of image datasets necessitate efficient storage, retrieval, and processing solutions. At present, the exchange of image data often relies on physical harddrives, institutional storage services, cloud storage tools like Dropbox, or occasionally public shared image databases combined with ontologies for annotation. However, these methods can be cumbersome and may not fully address the requirements for seamless collaboration and data sharing.

Furthermore, diverse research teams and institutions often use different imaging modalities, file formats, and analysis pipelines, which can create hurdles in the seamless exchange of data and knowledge (Moore et al., 2021). Compounded by the mandate from organizations such as the National Institutes of Health (NIH) requiring all data associated with a publication to be made available, the traditional desktop-based computation model, where data is uploaded as a final step, can pose challenges especially (thought not exclusively) when personnel may turn over during the publication process, making original source files harder to trace. The current single-user, single-desktop paradigm that dominates much of the current bioimage analysis landscape can hinder collaboration, as it requires specialized software and hardware resources that may not be universally available to all researchers. In contrast, a browser-based model, wherein data would be uploaded as an initial step, may offer a more seamless, efficient, and inclusive workflow.

In this paper, we explore the prospects for moving beyond the desktop and embracing web-based approaches to overcome these challenges and enhance the practicality of bioimage analysis for researchers worldwide.

## 2 Current approaches to bioimage analysis collaboration

**FIGURE 1:**
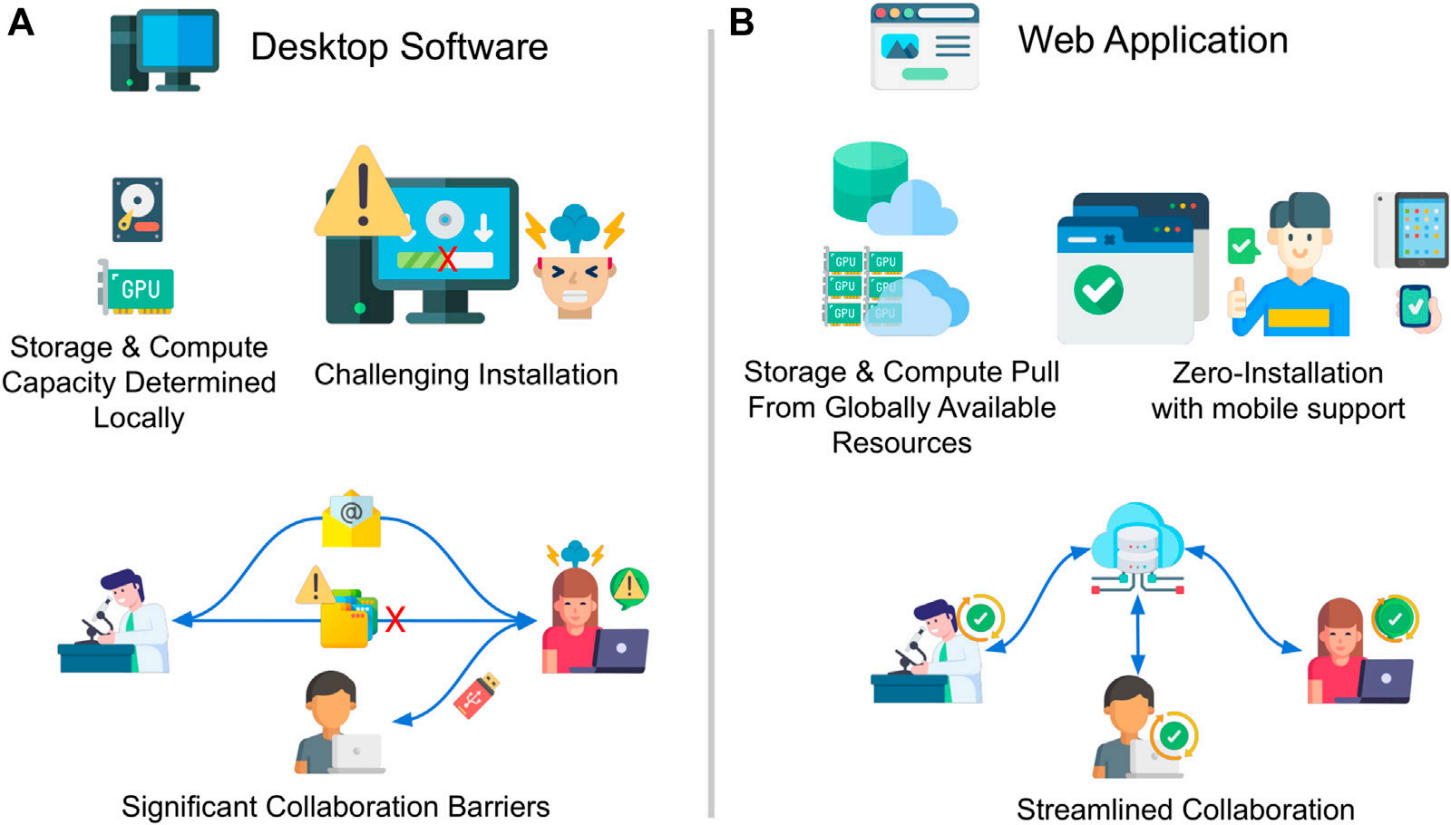
Desktop vs. Web bioimage analysis tools. Comparison of **(A)** conventional desktop-based bioimage analysis software with **(B)** the emerging web-based bioimage analysis software. When using conventional software, one must work to handle installation of software on a local computer, the hardware requirements, and the difficulties in collaboration and reproducibility due to varying software versions and platform dependencies. Web-based tools, while they have their own disadvantages, have significantly improved ease of access, reduced computational requirements for the end user, and enhanced collaboration through shared resources and data. The figure emphasizes the potential benefits of transitioning from desktop to web-based bioimage analysis tools in life sciences research. Image credit: Flaticon.com.

Conventional methods of sharing image files and analysis workflows often involve sending files via email, using cloud storage tools like Dropbox or Google Drive or sending physical hard drives via regular mail. However, these methods can be inadequate for handling large datasets shared among many users (Figure 1). Dedicated image data servers, such as OMERO (Allan et al., 2012), have been developed to address this issue by providing more efficient storage and management of large-scale datasets.

For public access, especially after publication, researchers commonly use shared databases such as the Image Data Repository (IDR) (Williams et al., 2017), the BioImage Archive (Hartley et al., 2022) and The Cell Image Library (Ellisman et al., 2021). These platforms facilitate the exchange of information and promote interoperability between analysis tools by providing standardized metadata and file formats. Ontologies, such as the EDAM Bioimaging Ontology (Kalaš et al., 2020), are also used for standardization of metadata, which greatly facilitates the exchange of information and helps the interoperability between analysis tools. The use of standardized file formats and metadata enables more efficient data sharing and collaboration while reducing the need to move data around.

Overall, current approaches to bioimage analysis collaboration rely on a combination of conventional file-sharing methods, dedicated image data servers, shared databases, and ontologies for metadata standardization. These methods often require moving data around, which is why they rely on standardized file formats for better compatibility and efficiency.

As we transition towards a more connected research environment, a growing number of researchers are uploading their data to cloud servers. This includes not only global services like AWS and GCP but also private institutional servers or inter-institutional servers connected via high-speed fiber networks, such as the JANET network in the UK (British Library. Research and Development Department, 1990). Such networks offer high-speed connections and substantial storage capacity, allowing local storage to serve merely as a buffer, providing a more sustainable storage solution.

Despite this shift, the current bioimage analysis software ecosystem remains primarily desktop-centric (Levet et al., 2021), necessitating users to download either complete images or data chunks to their local machines for analysis. This approach conflicts with the pricing models of many cloud providers, which typically offer inexpensive or free uploading and storage but impose more substantial fees for downloading data. This discrepancy underlines the necessity of transitioning to web-based tools, which can conduct analyses directly on cloud-stored data.

Comparison of a) conventional desktop-based bioimage analysis software with b) the emerging web-based bioimage analysis software. When using conventional software, one must work to handle installation of software on a local computer, the hardware requirements, and the difficulties in collaboration and reproducibility due to varying software versions and platform dependencies. Web-based tools, while they have their own disadvantages, have significantly improved ease of access, reduced computational requirements for the end user, and enhanced collaboration through shared resources and data. The figure emphasizes the potential benefits of transitioning from desktop to web-based bioimage analysis tools in life sciences research.

## 3 Web-based opportunities for bioimage analysis

Web-based bioimage analysis offers a promising alternative to conventional desktop-based analysis software. This approach encompasses native web tools as well as cloud-utilizing tools to enable researchers to initiate and carry out image analysis entirely through a web interface.

Native web tools are applications built specifically for the web, allowing users to perform tasks such as image visualization, processing, and analysis directly within their web browsers (Ouyang et al., 2019; Manz et al., 2022). Accessing web resources encompasses the use of both native web tools and cloud-based applications, which leverage online databases, libraries, and repositories to enhance bioimage analysis workflows. For example, OMERO servers are used by institutions for managing image databases stored on servers (Allan et al., 2012). Cloud resources enable researchers to offload computationally intensive tasks to remote servers, providing scalable computational power, storage, and specialized hardware for a wide range of applications. As an example, CDeep3M (Haberl et al., 2018) provides a plug-and-play cloud based deep learning solution for image segmentation of light, electron and X-ray microscopy.

One notable advantage of web-based tools is the ability to exchange data and analysis workflows through clickable URLs instead of explicitly downloading and uploading image files (Williams et al., 2017; Ouyang et al., 2019). This feature greatly improves user experiences, especially when working with tissue scans and large image files, as it simplifies data sharing and allows for streamlined collaboration among researchers.

Web-based bioimage analysis has the potential to address many of the limitations associated with the single-user, single-desktop paradigm by offering a more flexible, accessible, and collaborative platform for researchers. In the subsequent sections, we will discuss the benefits, existing tools, and challenges related to web-based bioimage analysis, as well as how the research community can transition from desktop-based to web-based analysis tools.

## 4 Benefits of web-based bioimage analysis

**FIGURE 2:**
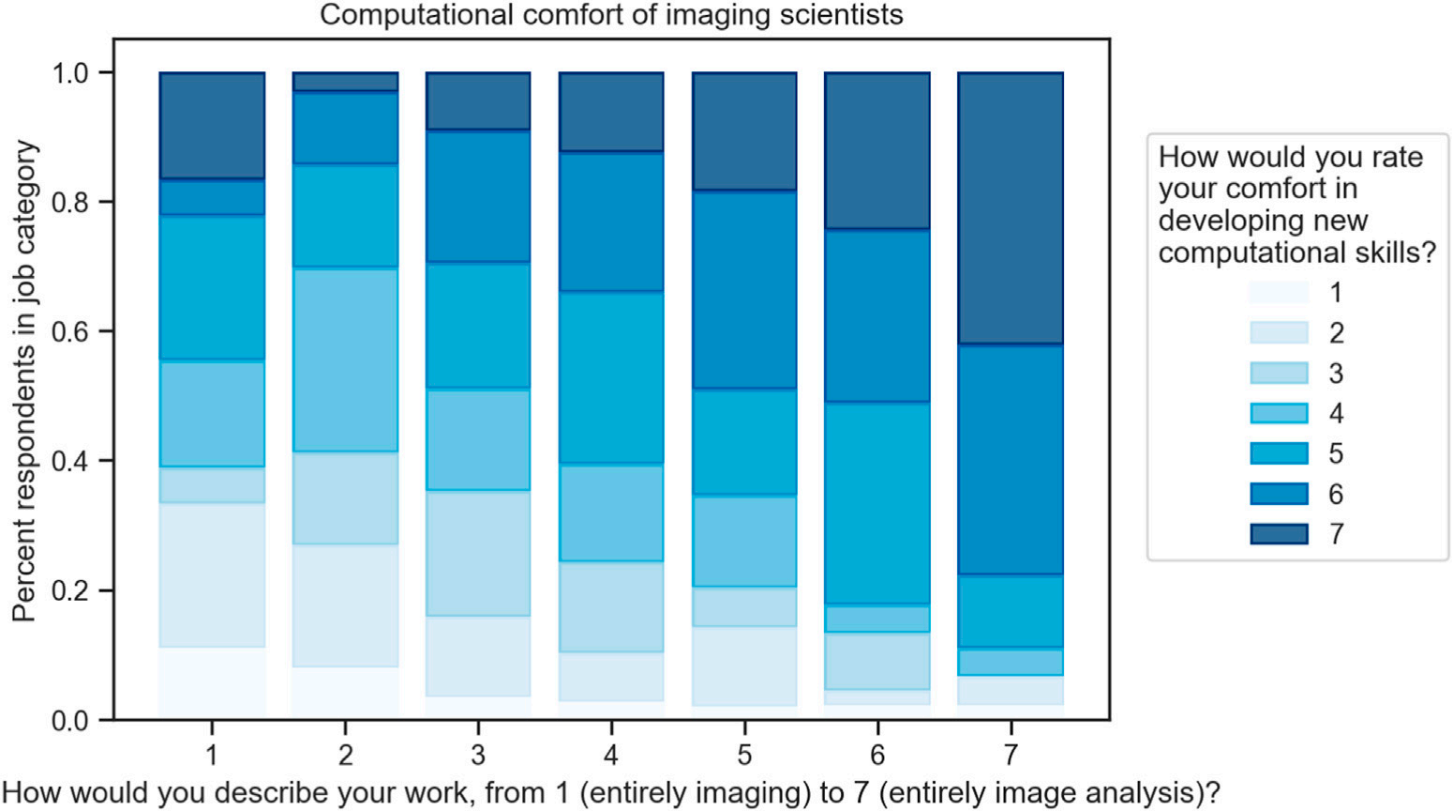
Computational comfort of scientists in a variety of work types. Approximately 500 respondents who use scientific imaging in the life or physical sciences were asked to describe their work on a scale from “entirely imaging” and “entirely image analysis”; in parallel, they were asked to report their comfort developing new computational skills. While the highest comfort levels are present in all groups, they are more common in users who spend more time performing analysis, and the lowest levels of comfort are represented almost entirely in the lower bins of computational work level. Data are normalized within each work level by the number of responses in that level: 1:18, 2:63, 3:88, 4:185, 5:59, 6:45, 7:45 Data from (Sivagurunathan et al., 2023).

Web-based bioimage analysis offers a range of benefits that can improve the overall user experience, accessibility, and efficiency of biological research. One notable advantage is the lower computational comfort required for researchers who primarily focus on imaging rather than analysis (Figure 2). By offering user-friendly interfaces and streamlined workflows, web-based tools can help bridge the gap between image acquisition and data analysis, making it easier for researchers with limited computational expertise to engage in meaningful bioimage analysis.

An analysis of the Scientific Community Image Forum (Rueden et al., 2019) (https://image.sc), a popular online expertise sharing resource for bioimage analysis, reveals that a significant portion of discussions revolve around installation issues. Analysis performed for this paper showed that approximately 7%–8% of recent posts in the forum (approximately 1 in every 12–14) contain the word “install”. Furthermore, installation topics come up at least once in ∼19% of the ∼500 most recently posted threads, and are mentioned in the opening post 9.9% of the time. This suggests that about 1 in 10 threads in the forum are likely started due to an installation issue, and 1 in 5 threads end up discussing it at some point.

The high frequency of installation-related discussions highlights the challenges researchers face when setting up and using traditional desktop-based bioimage analysis tools. By contrast, web-based tools can significantly alleviate these challenges by moving the installation burden entirely to the developer or by offering easy installation and updates, eliminating the need for complex configuration processes, and ensuring cross-platform compatibility.

Transitioning to web-based bioimage analysis offers numerous advantages over the traditional desktop approach, beyond ease of installation, sharing, and collaboration, allowing researchers to efficiently transition from testing selective prototyping workflows on small data subsets (e.g., the CellPose (Stringer et al., 2021) web application at https://www.cellpose.org/) to applying proven methods to full-scale datasets. Notably, it addresses the challenge of having to download entire datasets to local computers for visualization or processing using desktop software. Instead, web-based tools allow for the processing of data directly in the cloud, transmitting only necessary portions to the user’s browser for review. This not only optimizes the use of available bandwidth but also aligns well with the pricing models of many cloud services. In effect, this approach allows us to harness the sustainable storage capacity and computational power of the cloud to enhance the efficiency and effectiveness of bioimage analysis.

## 5 Existing tools for web-based bioimage analysis

Various tools have now been developed to facilitate web-based bioimage analysis. These tools can be categorized into scalable storage image formats, browser-based visualization and computing, and cloud or server-based image analysis platforms.

Scalable storage image formats, such as OME-NGFF (Moore et al., 2021), enable remote visualization in tools like Fiji (Schindelin et al., 2012)’s MoBIE (Pape et al., 2023). OME-NGFF is suitable for storing massive datasets in chunks and serving them using object storage, such as S3 or HTTP servers. Browser-based visualization tools, including Kitware’s ITK VTK Viewer (McCormick et al., 2023), Viv (Manz et al., 2022), OpenSeadragon (Github, 2023), TissUUmaps (Solorzano et al., 2020), and webknossos (Boergens et al., 2017), offer easy-to-use and performant visualization due to their utilization of browser GPU standards, such as WebGL or WebGPU. These tools are ideally suited for accessing chunked storage formats like OME-NGFF or OME-Zarr (Moore et al., 2023).

For initial exploration and selective prototyping workflows, browser-based computing tools are of great value, browser-based computing tools, such as Piximi (Goodman et al., 2021), ImJoy and ImageJ.JS (Ouyang et al., 2019), can perform computation locally in web browsers. While useful and secure, since computation happens locally, their performance is limited by memory and compute constraints (e.g., 4 GB memory per tab in Chrome browser) and lack of direct access to GPU accelerators, such as CUDA.

In contrast, for applying workflows to large-scale data, cloud or server-based image analysis platforms often offer a scalable solution. Tools, such as ImJoy (with Jupyter (Kluyver et al., 2016)/BinderHub (Project Jupyter et al., 2018) backend), DeepCell Kiosk (Bannon et al., 2021), Terra (Broad Institute of MIT and Harvard, Microsoft, and Verily), Galaxy (Galaxy Community et al., 2022), KNIME (Berthold et al., 2007), CDeep3M (Haberl et al., 2018), Bisque (Kvilekval et al., 2010), BAND (EMBL, 2023), Cytomine (Marée et al., 2016), Colab notebooks, and ZeroCostDl4Mic (von Chamier et al., 2021), provide a more scalable way of storing, sharing, and processing image data. These platforms are especially helpful for AI-based analysis pipelines, given the ongoing trend towards foundation models (Bommasani et al., 2021), such as Segment Anything (Kirillov et al., 2023), diffusion models (Ho et al., 2020), or transformer-based large language models for generating code (Chen et al., 2021) (e.g., in Python) for automated image analysis. In cloud environments, containers are often used to create reproducible environments, orchestrated by cluster software like Kubernetes.

## 6 Challenges in implementing web-based bioimage analysis

While web-based bioimage analysis offers substantial benefits, transitioning to this model comes with numerous inherent challenges that require strategic navigation. For instance, browser-based tools might encounter performance limitations due to memory and computational constraints. Furthermore, privacy concerns may arise when users are hesitant to upload sensitive image data to servers managed by third-party service providers. Nevertheless, hybrid computing models, such as the one demonstrated in the Segment Anything demo application, provide promising solutions. These models blend the flexibility of browser-based computation with the robust power of server-side computation. But this transition is not without its obstacles.

One of the most significant hurdles is the handling of massive image datasets that are fundamental to bioimage analysis. Uploading these considerable amounts of data to the cloud is not only a time-consuming task but also presents logistical difficulties, even with high-speed networks within the institution/university. Moreover, conventional methods, which necessitate periodic downloading of data, can lead to additional costs and potential delays.

There are, of course, additional challenges to overcome when implementing these solutions.

1) Algorithmic challenges: Algorithms need to be tailored to fit the browser environment, which is typically single-threaded and memory-constrained. Optimization is often required to run efficiently within the browser. Remote servers can be used to complement storage and compute limitations.
2) Cross-language and algorithmic accessibility: With WebAssembly, algorithms implemented in languages such as C/C++ can be compiled to run in the browser. However, not all languages are readily compatible with web-based platforms, requiring additional effort for adaptation.
3) Deep learning integration: Modern web browsers support deep learning frameworks like TensorFlow.js, some even leveraging WebGPU. However, due to browser constraints, remote servers or local compute engines (e.g., Jupyter servers) are often necessary for more demanding tasks.
4) Handling big data: Although browsers can store data, they are better suited as clients for data handling when dealing with large datasets. Efficient data management strategies are needed to accommodate big data in web-based bioimage analysis.
5) Performance optimization: Striking a balance between limited local compute power and storage in the web browser, and reducing data transmission with remote servers, is crucial. Utilizing browser-based compute engines like WebWorkers and WebGPU can help optimize performance.
6) Efficient data transfer: Data compression techniques and browser-based compute can help reduce latency and data transmission. Efficient data transfer methods are essential for maintaining responsive web-based bioimage analysis tools.
7) Security concerns: In addition to conventional security measures such as encryption, edge computing using the browser can help alleviate privacy concerns. Ensuring data security and user privacy is vital when implementing web-based bioimage analysis tools.
8) Funding challenges: It may be challenging to gauge the cost needed to support the server infrastructure when hosting one’s own server, especially if wide adoption increases traffic. Most projects are funded on short-term grant models, making it challenging to secure the future of a server long term, especially as funding mechanisms for maintenance are scarce relative to those for tool creation.
9) Additional challenges: Usability, maintenance, and support for web-based tools are also important considerations. Ensuring that tools are user-friendly and well-documented, while providing ongoing support and updates, will contribute to the success of web-based bioimage analysis platforms.

Addressing these challenges requires a collaborative effort from the research community and developers to create robust, efficient, and user-friendly web-based bioimage analysis tools.

## 7 Transitioning from desktop-based to web-based analysis tools

The transition from conventional desktop-based bioimage analysis to web-based platforms heralds a significant evolution in bioimage analysis. This shift necessitates a fundamental change in the traditional approach, where data, computation, and user interface (UI) reside together with the user. In the web-based model, data and computation are hosted remotely, leaving only the UI with the user. This setup means that only a small portion of raw data and relevant statistics need to be transferred to the user for inspection, validation, and workflow development.

To address data transmission issues, it is beneficial to design cloud and web-native software that aligns with cloud providers’ pricing models, keeping most data in the cloud. Techniques like pyramid-like chunked image viewers supporting OME-NGFF (e.g., Viv, itk-vtk-viewer) could serve to transfer only necessary data portions to the user, reducing the need for full dataset downloads.

Several key considerations could further facilitate this transition.

1) Adapting Current Tools for Web-Based Use: Existing bioimage analysis tools should be adapted to operate within the web environment. This process may require code refactoring, optimizing algorithms for browser-based execution, and integrating tools with web-based platforms and services. For desktop tools to run remotely in a cloud environment, “headless” execution and batch processing capabilities with a scripting interface are crucial. New software should adopt distributed software design principles, separating the user interface from computational components. This allows the UI to run in the browser while computations occur on a remote server, achieved by Remote Procedure Calls.
2) Development of Adaptable Compute Engines: An essential step towards effective web-based bioimage analysis is the creation of adaptable compute engines capable of adjusting to various cloud environments. This approach acknowledges the advent of “Sky Computing,” which foresees a more collaborative and interconnected cloud environment (Stoica and Shenker, 2021). A community-driven effort is needed to create a compatibility layer on top of diverse clouds, whether private or public, which could enable greater interoperability for developers.
3) Coding Efforts: Transition to web-based tools often requires learning new programming languages and frameworks like JavaScript, WebGL/WebGPU, or Unity. However, with tools like WebAssembly and web compilers, developers can port existing libraries to run in the browser, like ImageJ.JS that was compiled from Java to JavaScript with a compiler named CheerpJ. Developers must also familiarize themselves with cloud-based services and remote computing resources to leverage scalable storage and computation.
4) Community Involvement: Successful transition to web-based bioimage analysis tools calls for substantial community involvement, encompassing researchers, developers, and industry partners. Collaboration is pivotal for the development and maintenance of web-based tools, sharing best practices, and tackling common challenges. An open-source development approach, along with sharing code, data, and resources, fosters a more inclusive and efficient research community.

By addressing these areas, the research community can collectively foster a new generation of powerful, accessible, and collaborative web-based bioimage analysis tools, surmounting the limitations of traditional desktop-based methods.

## 8 Conclusion

Web-based bioimage analysis has the potential to profoundly transform the methodologies of biological research. By transitioning from desktop to the web, it is possible to offer improved accessibility, scalability, and reproducibility, ultimately democratizing resources for researchers globally and enhancing collaboration across various domains.

Overcoming the hurdles associated with implementing web-based bioimage analysis tools requires the collaborative effort of the research community, developers, and industry partners. By adapting and optimizing existing tools and approaches, embracing new programming languages and frameworks, and fostering community involvement, we can collectively advance towards a web-based paradigm that is mutually beneficial.

As we shift from desktop-based to web-based analysis tools, the maintenance of user experience, performance optimization, and security is of utmost importance. Developing efficient, user-friendly, and secure web-based bioimage analysis platforms is a shared responsibility. We must continue to innovate, share experiences, and foster a culture of open dialogue to facilitate this progress.

In conclusion, web-based bioimage analysis stands at the precipice of the future of biological research. By cooperating to overcome challenges and build robust, accessible tools, we can unravel the full potential of web-based bioimage analysis, paving the way for revolutionary discoveries in the life sciences.
